# Flat mount preparation for whole-mount fluorescent imaging of zebrafish embryos

**DOI:** 10.1242/bio.060048

**Published:** 2023-07-20

**Authors:** Joseph Frommelt, Emily Liu, Afraz Bhaidani, Bo Hu, Yuanyuan Gao, Ding Ye, Fang Lin

**Affiliations:** Department of Anatomy and Cell Biology, Carver College of Medicine, University of Iowa, Iowa City, IA, 52242, USA

**Keywords:** Flat mount preparation, Deyolking, Confocal imaging, Zebrafish embryos

## Abstract

The zebrafish is a widely used model organism for biomedical research due to its ease of maintenance, external fertilization of embryos, rapid embryonic development, and availability of established genetic tools. One notable advantage of using zebrafish is the transparency of the embryos, which enables high-resolution imaging of specific cells, tissues, and structures through the use of transgenic and knock-in lines. However, as the embryo develops, multiple layers of tissue wrap around the lipid-enriched yolk, which can create a challenge to image tissues located deep within the embryo. While various methods are available, such as two-photon imaging, cryosectioning, vibratome sectioning, and micro-surgery, each of these has limitations. In this study, we present a novel deyolking method that allows for high-quality imaging of tissues that are obscured by other tissues and the yolk. Embryos are lightly fixed in 1% PFA to remove the yolk without damaging embryonic tissues and are then refixed in 4% PFA and mounted on custom-made bridged slides. This method offers a simple way to prepare imaging samples that can be subjected to further preparation, such as immunostaining. Furthermore, the bridged slides described in this study can be used for imaging tissue and organ preparations from various model organisms.

## Teaser

**Summary*:*** A new deyolking method for zebrafish embryos enables high-resolution confocal imaging of deep tissues, offering a simple and effective technique for biomedical research.

## INTRODUCTION

Zebrafish are a tropical freshwater species and a powerful vertebrate animal model for studying developmental and cellular processes (Choi et al., 2021; Stainier et al., 2017). As vertebrates, they have a high level of genetic conservation with humans (Howe et al., 2013; Postlethwait et al., 1998), making them a valuable tool for understanding the complex cellular processes underlying human development and diseases. And compared to mammalian models, zebrafish have many advantages: they are easy to maintain, inexpensive, and able to produce a large number of embryos that develop rapidly, which allows for high-throughput research such as genetic screening and drug testing. Zebrafish embryos are particularly valuable because they are mostly transparent, enabling high-resolution imaging experiments that can use various labeling techniques, including staining, immunofluorescence, and transgenic markers (Bruneel et al., 2015; Megason and Fraser, 2003; Weber and Koster, 2013). Furthermore, the availability of advanced genetic tools in zebrafish allows for precise manipulation of gene expression in specific tissues at certain times (Albadri et al., 2017; Amsterdam and Hopkins, 2006; Hwang et al., 2013; Kalvaityte and Balciunas, 2022).

Although zebrafish embryos are transparent and suitable for many imaging applications, visualizing tissues and organs located deep within the embryo becomes challenging as the embryo forms multiple layers of tissue during development (Kimmel et al., 1995). By the end of gastrulation, which occurs around 12 hours post-fertilization (hpf), the embryo has established three distinct germ layers: the ectoderm, mesoderm, and endoderm (the deepest layer), which cover the entire yolk sac. During segmentation, the embryonic axis forms at the dorsal region of the embryo, and the embryonic trunk becomes thicker and longer, wrapping around the yolk. By 24 hpf, the embryo elongates further as many organs, including the gut tube and associated organs, start to develop. By 48 hpf, most organs have completed development. At this stage, the yolk continues to shrink but is still located beneath the trunk region of the embryo. The formation of multiple layers of tissue presents a challenge for imaging deeper structures such as the heart and the digestive system due to the slight absorbance of light by each tissue, which obscures the target region. The yolk is even more difficult for light to pass through, so tissues located between the yolk and other tissue layers, such as endoderm, are the most challenging to image.

To image the deep tissues in zebrafish embryos, several methods are available, including two-photon live imaging (Abu-Siniyeh and Al-Zyoud, 2020; Renninger and Orger, 2013), cryosectioning, vibratome sectioning, and microsurgery. However, two-photon microscopes are not commonly available in many research institutes, which limits the use of this technique. Cryosectioning and vibratome sectioning can expose the deep tissues but are highly sensitive to the specific angle and region of the cut, and landmark features are necessary to determine where each section was obtained from within the whole embryo (Ye et al., 2015; Yin et al., 2010). Additionally, acquiring all the necessary sections to gather information on the entire tissue or organ structure being analyzed can be a challenge. Although microsurgery can dissect some tissues in zebrafish embryos, such as the heart (Yang and Xu, 2012), this technique can be difficult to use for delicate tissues such as the endoderm. At present, the field would benefit from an accessible method of preparing samples for imaging deeply situated tissues.

Here, we present a novel deyolking method for zebrafish embryos that enables imaging of deeper tissues that are closer to the yolk. This technique involves carefully removing the yolk without damaging the embryonic tissue after lightly fixing it with 1% paraformaldehyde (PFA), followed by refixing the embryos in 4% PFA and mounting them on custom-made bridged slides. This method offers a straightforward approach to preparing large numbers of samples that can be subjected to further preparation, such as immunostaining. Additionally, the bridged slides described in this study can be modified and used for imaging sample preparations from various animal models, making this technique broadly applicable for a wide range of sample types.

## RESULTS

### Collecting the zebrafish embryos

Zebrafish embryos are obtained through the crossing of sexually mature fish, which can be achieved in various ways. The following procedures outline the practices followed in our fish facility. On the day before embryo collection, adult zebrafish are prepared for mating between 3–8 pm. This is done by placing up to 1–2 males and 1–2 females in breeding tanks equipped with egg filters and dividers that separate the males and females. The dividers are used to control the timing of when the fish are allowed to cross and produce embryos. To obtain embryos, the dividers in the breeding tanks are removed to allow the fish to spawn. The containers are periodically checked to see if zebrafish have laid any embryos. Embryos are collected at 5–10-min intervals to ensure minimal variation in age due to multiple spawns.

To collect the embryos, the water containing the embryos is poured through a strainer. The embryos are then washed into a 10 cm petri dish filled with egg water. Unfertilized and non-viable embryos, as well as debris, are removed from the dish. The remaining embryos are incubated at 28.5°C. To avoid overcrowding, no more than 50 embryos are placed in each dish. Throughout the day, the embryos are examined to monitor their developmental progression according to the zebrafish standard staging series (Kimmel et al., 1995). For harvesting embryos at 24 and 48 hpf, the embryos are grown at 28.5°C and fixed accordingly. To slow down development, embryos can be transferred to lower temperatures, such as 20–22°C, when they reach the sphere stage, which occurs approximately 4 hpf. Very young embryos (before the sphere stage) should not be grown at temperatures below 22°C. Early embryogenesis is sensitive to low temperatures, and embryos may fail to develop properly or die under such conditions.

The timing for harvesting and transferring embryos to different temperatures, as well as the specific incubation temperatures, can be optimized to obtain embryos at target stages. Table 1 provides information on the growing conditions used in our experiments, including the estimated time for removing the dividers, the stage of embryos to be transferred to different temperatures overnight, and the estimated stages on the following day.

**Table 1.**
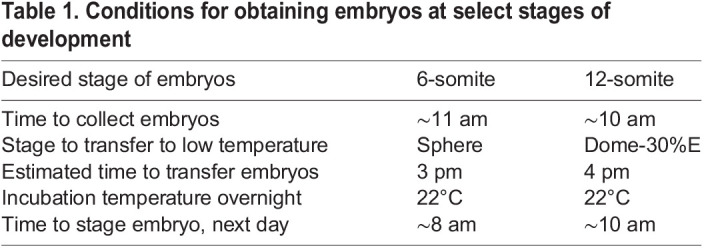
Conditions for obtaining embryos at select stages of development

### Fixing the zebrafish embryos

**Fig. 1.**
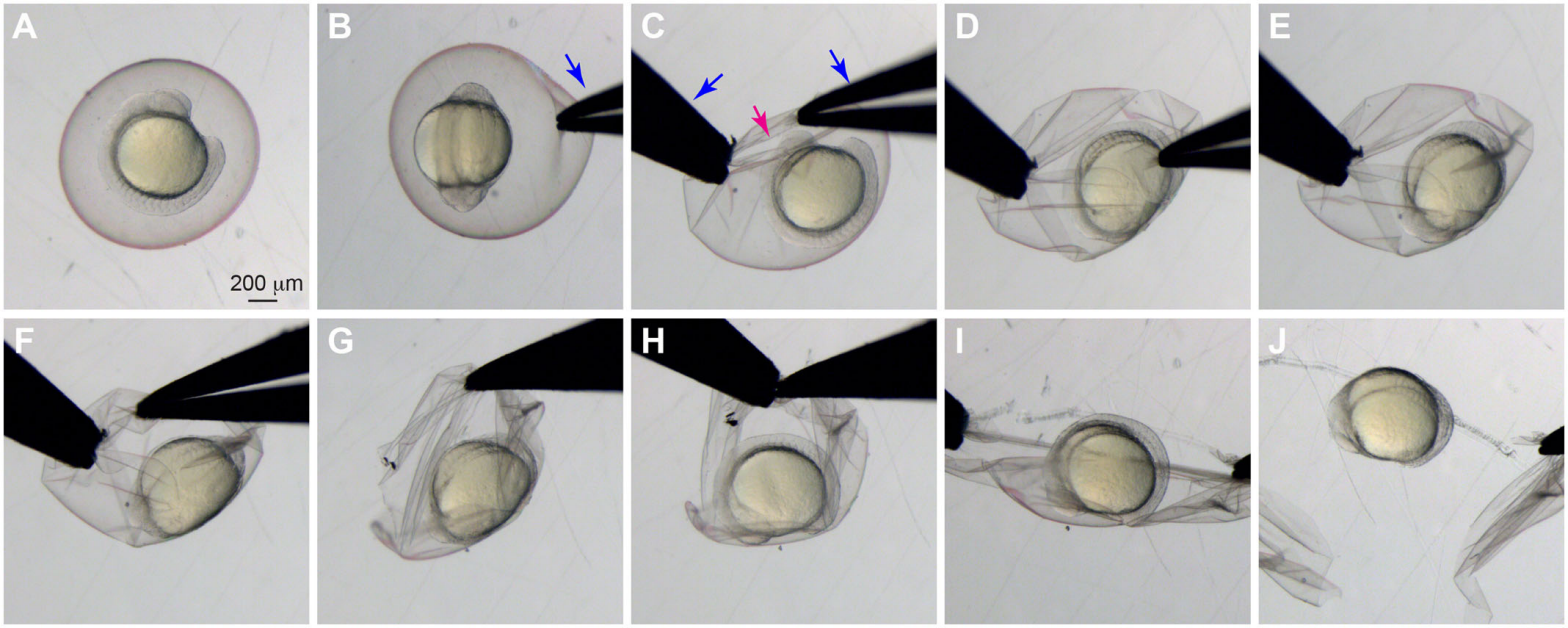
**Manual dechorionation of live zebrafish embryos.** Bright-field images illustrating the dechorionation process. (A) A live embryo before dechorionation. (B) A pair of forceps is used to gently pinch the chorion. (C) A second pair of forceps is used to pinch the chorion near the first pinch and to gently pull it apart. (D) The hole is made bigger, but not so much that the chorion collapses inward and squeezes the embryo. (E,F) Forceps are repositioned as needed to avoid squeezing the embryo. (G,H) Repositioning multiple times may be necessary. (I) Once the hole is large enough, the chorion is pulled apart without it collapsing inward and squeezing the embryo. (J) The embryo after dechorionation. Magenta arrow, hole in the chorion; blue arrows, forceps.

Before fixing embryos at the desired developmental stages, it is necessary to remove their chorions (a process called dechorionation) to allow the fixative (1% PFA) to properly penetrate the embryos. There are multiple methods to dechorionate the embryos. Young embryos are fragile and can be damaged by the chorion if squeezed. Therefore, the following procedure is commonly used in our laboratory, particularly for embryos at a young age (before 24 hpf): we use two pairs of sharp forceps to make a small tear in the chorion and then gently pull to create a larger hole through which the embryo can slip out of the chorion (Fig. 1). One pair of forceps is used to pinch the chorion gently (Fig. 1B), while the other pair of forceps performs an additional shallow pinch near the first pinch and gently pulls the chorion to create a small hole (Fig. 1C). The size of the hole is gradually increased by repositioning one pair of forceps to pull the hole open from a different direction (Fig. 1D-H). Eventually, the hole will be large enough for the embryo to fall out (Fig. 1I-J). In the case of older embryos, repositioning the forceps is often unnecessary, and the chorion can be directly pulled apart to create a hole big enough for the embryo to fall out.

Carefully transfer the dechorionated embryos using a glass pipette into 2 ml microcentrifuge tubes, ensuring that no more than 20 embryos are placed in each tube. Properly label each tube. Before adding the appropriate fixative, remove as much egg water from the microcentrifuge tubes as possible using the glass pipette. It is recommended to exchange any buffers in tubes containing embryonic samples under a dissecting microscope, as it is easier to detect accidental removal of samples under magnification.

**Fig. 2.**
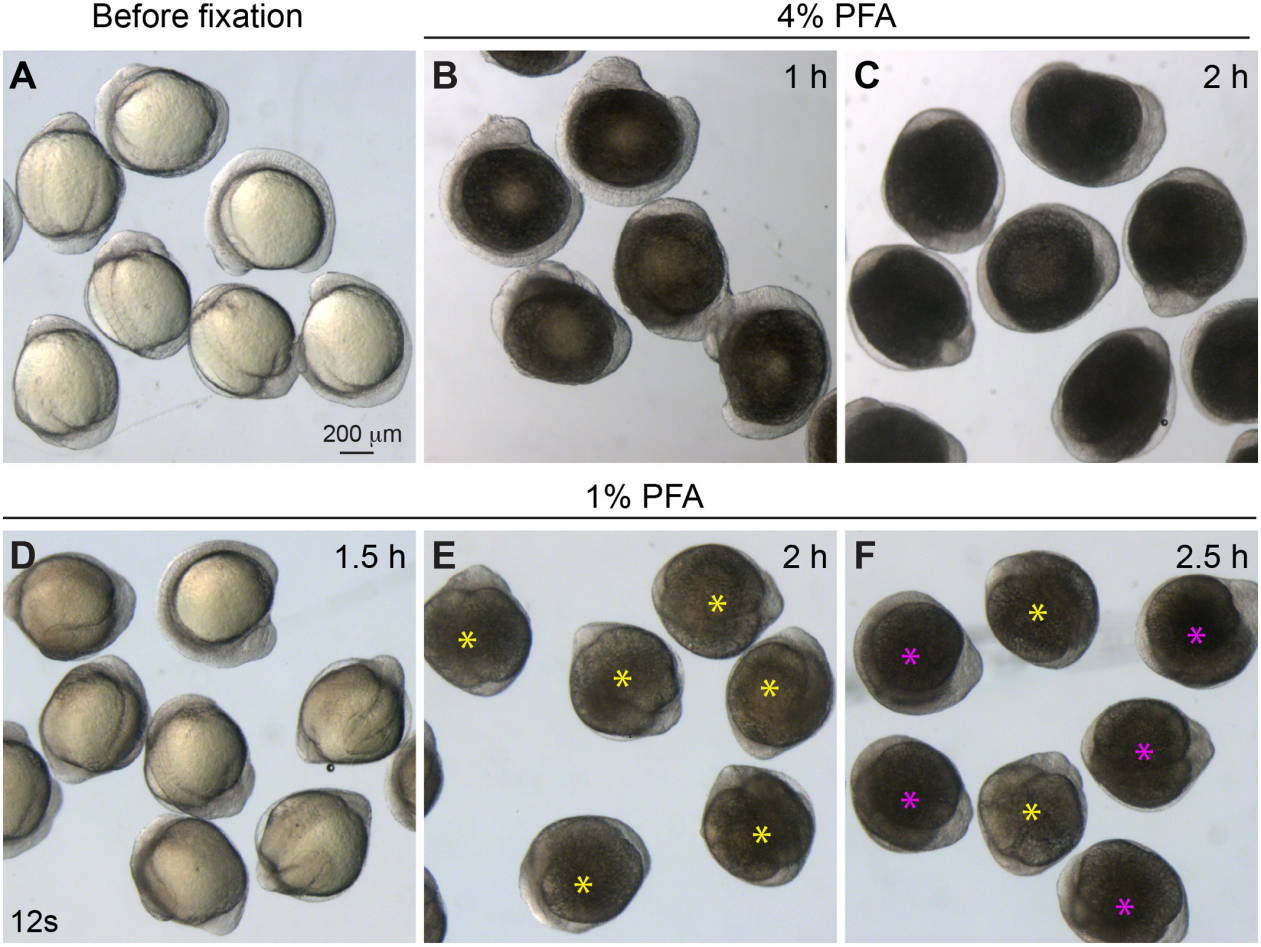
**Embryo fixation under different conditions.** Bright-field images of embryos at 12 somites. (A) Live embryos before fixation. (B-C) Embryos fixed with 4% PFA for 1 h (B), and 2 h (C), both over-fixed. (D-F) Embryos fixed with 1% PFA for 1.5 h (D, under-fixed), 2 h (E, optimally fixed, yellow asterisks) and 2.5 h (F, some are over-fixed, magenta asterisks; some are optimally fixed, yellow asterisks).

For various applications, such as *in situ* hybridization and immunostaining, 4% PFA is commonly used to fix zebrafish embryos. Typically, this is done at room temperature for 2–4 h or at 4°C overnight (Hu et al., 2021; Thisse and Thisse, 2008). However, we have observed that even a 1-h fixation with 4% PFA can lead to over-fixation of the yolk cells, resulting in a dark grey or black coloration of the yolk (Fig. 2B,C). Consequently, the yolk can tightly adhere to the embryonic tissues, making it challenging to remove. To identify an appropriate fixation condition for deyolking, we conducted experiments involving different concentrations of PFA, fixation durations, and temperatures (Fig. 2). Our findings demonstrated that using 1% PFA for 2 h at room temperature or overnight at 4°C yielded the best outcomes, with yolk cells exhibiting a golden grey color (Fig. 2E). Under these conditions, the yolk cells can be easily removed using the method described below. However, over-fixation can occur with durations exceeding 2 h. In some embryos, the yolk appeared to be dark grey (Fig. 2F, indicated by magenta color). On the other hand, under-fixation can result from shorter fixation times, such as 1–1.5 h, leading to light grey yolk cells (Fig. 2D). These under-fixed embryos tend to have excessively sticky and fragile embryonic tissues.

For the deyolking procedure, add approximately 1.5 ml of freshly prepared 1% methanol-free PFA to each tube. Gently invert the tubes a few times before placing them in a dark box to prevent bleaching of fluorescent labeling. Place the box on a rotator set at low speed for a duration of 2 h at room temperature or overnight at 4°C. After fixation in 1% PFA, wash the embryos by removing the 1% PFA solution and adding PBST (0.01% Triton X-100) using a glass transfer pipette. Return the tubes to the dark box and place them on the rotator for at least 1 min. Repeat this washing step twice, ensuring that the embryos are washed with PBST three times in total.

### Deyolking the zebrafish embryos

**Fig. 3.**
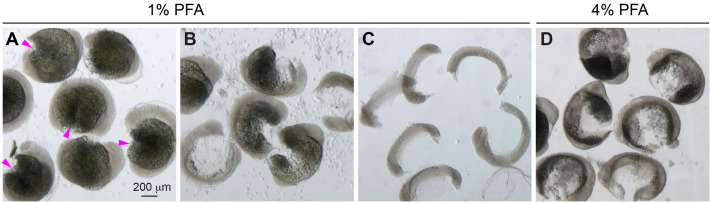
**Manual deyolking of embryos.** Bright-field images of manually deyolked embryos. (A) Embryos with pierced yolk sacs (magenta arrowheads). (B) Embryos with partially or almost-completely removed yolk cells. (C) Embryos with completely removed yolks. (D) Embryos fixed in 4% PFA still retained many yolk cells after aggressive and persistent pipetting.

After washing out the embryos from 1% PFA, the yolk is manually removed from the fixed embryos. Place the embryos in a nine-well depression dish (preferred, see Movie) or in a 35-mm glass dish with PBST (0.01% Triton X-100). Pierce the yolk sac using a pair of very fine forceps, taking care to avoid areas near the embryo (Fig. 3A, magenta arrowheads). It may be easier to pierce the yolk sac with the assistance of a second pair of forceps, which can hold the embryo steady by gently pushing against it to maintain its position while piercing the yolk sac. Pipette the embryos up and down in PBST using a glass transfer pipette until the yolk cells are sufficiently removed (Movie).

As yolk cells fill the dish and visibility decreases, transfer the embryos to a new well or dish with fresh PBST. Once an embryo no longer has yolk obscuring the region of interest, transfer it to another well or dish to prevent damage while continuing to pipette the remaining embryos. If the fixation is optimal, yolk cells can be easily removed without vigorous pipetting (Fig. 3B,C). However, in cases of over- or under-fixation, yolk cells may stick to the embryonic tissues even after vigorous pipetting (Fig. 3D). We observed that aggressive pipetting can easily damage the endoderm, which is located just above the yolk. But if the tissues of interest remain undamaged by such forceful movements, the embryos can be pipetted vigorously, and any remaining yolk cells can be manually removed as necessary using other hand tools such as an eyebrow or lasher, as described previously (Cheng et al., 2014).

After removing the yolk, transfer the deyolked embryos back to the microcentrifuge tube and carefully aspirate PBST. Due to the absence of the large yolk, the embryos are now lighter and more difficult to be seen, making them less likely to sink easily to the bottom of the tube. To prevent inadvertently removing deyolked embryos, it is recommended to keep the tubes in an upright position, allowing the embryonic tissues to gradually settle at the bottom of the tubes. Remove PBST against a contrasting background, ensuring good lighting conditions or using a dissecting microscope.

Following this, the deyolked embryos are refixed in 4% PFA as embryonic tissues are not properly fixed with 1% PFA. Refixation should be carried out at room temperature for 2 h or at 4°C overnight on a rotator set at low speed. After fixation, wash out the PFA with PBST using the same method described earlier. Repeat this washing step twice, resulting in a total of three washes with PBST. The embryos are now ready for immunostaining procedures or can be mounted for confocal imaging. Deyolked embryos refixed in 4% PFA can also be stored at 4°C in a dark location for up to one week.

### Immunostaining

It is important to note that different antibodies may require specific permeabilization and fixation methods. We have successfully used a list of antibodies with the described fixative method (refer to Table S1 for the complete list).

For permeabilization, two methods can be used. The first method is the ddH2O/Acetone method, which is preferred for detecting membrane proteins. To perform this method, the embryos are initially incubated in ddH2O for 5 min at room temperature. After removing the ddH2O, ice-cold acetone is added, and the samples are placed at −20°C for 7 min (or 5 min for embryos obtained before 12 hpf). Care should be taken to avoid overtreatment. Subsequently, the acetone is removed, and the embryos are washed in ddH2O. The washing times depend on the developmental stage of the embryos: two washes of 5 min each for embryos younger than 24 hpf, a 30-min wash for embryos obtained at 24 hpf, two 30-min washes for embryos obtained at 48 hpf, and four 30-min washes for embryos obtained at 72 hpf.

The second method for permeabilization involves using 0.5% Triton X-100 for a duration of 60 min. This method is particularly suitable for embryos before segmentation and for detecting non-membrane proteins, as it has been observed that acetone can damage embryos at a young age, while Triton X-100 has the potential to harm membrane proteins. It is crucial to select the appropriate permeabilization method based on the targeted proteins and their subcellular localization. Care should be taken to avoid excessive exposure to permeabilization agents to prevent any potential harm to the embryos or the proteins of interest.

After permeabilization, the embryos are rinsed twice with PBST (0.1% Triton X-100) and then transferred to a 24-well dish for immunostaining. Deyolked embryos, being lighter, may have difficulty sinking to the bottom of tubes and can be challenging to see. Hence, it is highly recommended to perform immunostaining in a 24-well dish. To improve visibility, placing a piece of dark paper at the bottom of the dish can be beneficial. Additionally, gently tapping the dish can assist the embryos in sinking to the bottom of the wells. When removing solutions from the wells, using a fine plastic transfer pipet with a small opening is convenient and effective.

Subsequently, the PBST is replaced with 500 μl of AB blocking buffer, and then the embryonic tissues are incubated at room temperature for 1 h. After the incubation period, the AB blocking buffer is removed, and the primary antibody, diluted in AB blocking buffer (300 μl per well), is added to the embryos. The embryos are then incubated at room temperature for 2–4 h or at 4°C overnight. To ensure optimal antibody performance, it is recommended to centrifuge both the stock and working solutions of all antibodies at maximum speed for 1 min to separate any particles that may affect the experimental results. Following the incubation with the primary antibody, the antibody solution is removed (the antibody can be saved and reused if stored at 4°C). The embryos are washed six times with PBST (0.1% Triton X-100)-DMSO at room temperature, with each wash lasting for 15 min. The incubation and washing processes are performed on a rotator set at a low speed.

Next, the secondary antibody solution in AB blocking buffer (300 μl per well) is added to the embryos in the 24-well dish. The dish is then placed in a dark box and the embryos are incubated at room temperature for 2–4 h or at 4°C overnight. After the incubation, the embryos are washed with PBST-DMSO, performing six washes for 15 min each at room temperature. If desired, the embryos can undergo staining with DAPI (0.2 mg/ml) for 15 min at room temperature and/or phalloidin (1:100) for 1–4 h at room temperature. Following staining, the embryos are washed again with PBST (0.01% Triton X-100), performing four washes for 5 min each at room temperature. Finally, the embryos are stored in PBST (0.01% Triton X-100) in a dark place at 4°C for further analysis or imaging.

### Mount deyolked embryos for confocal imaging

**Fig. 4.**
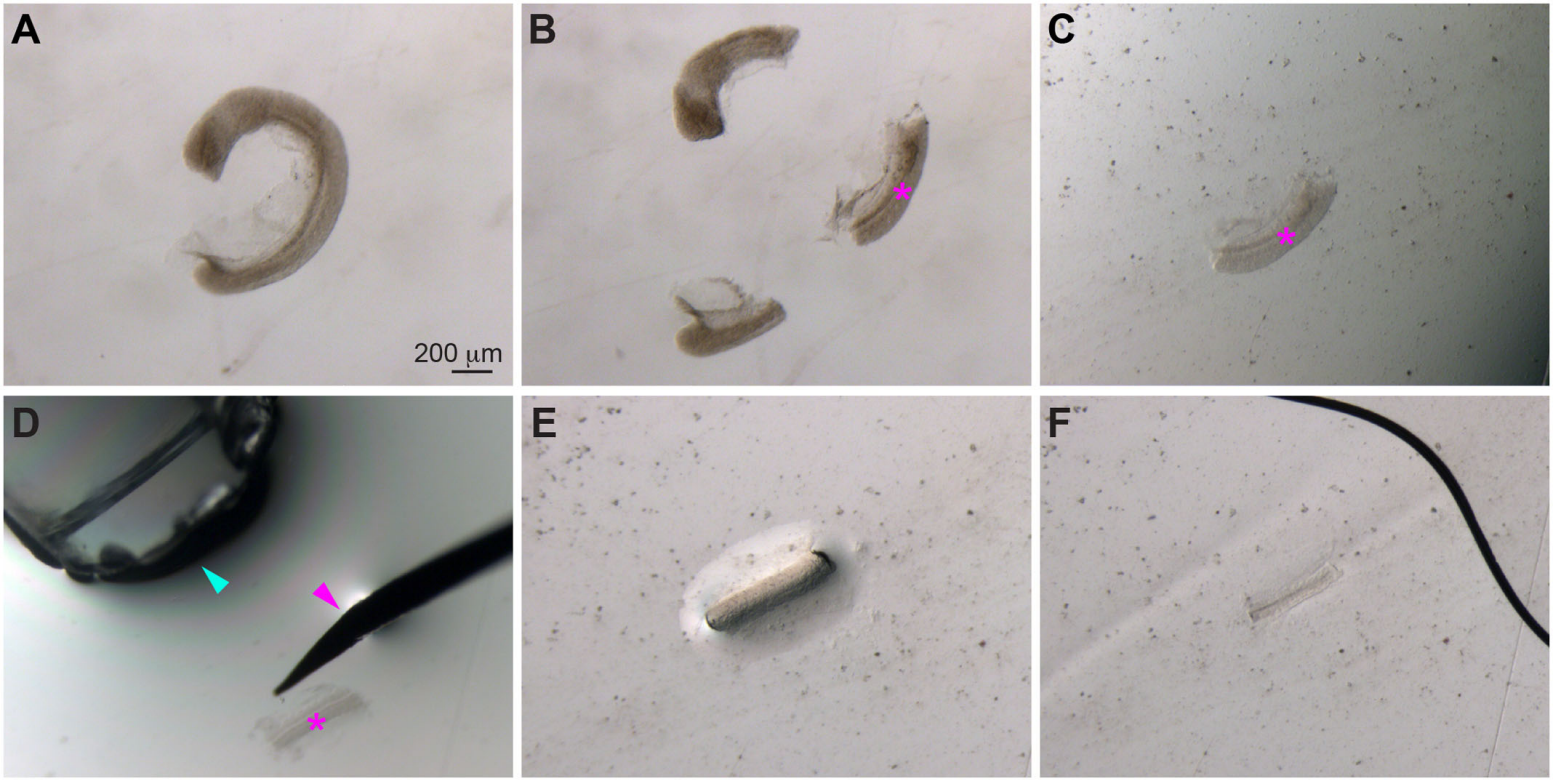
**Deyolked embryonic tissue mounted on bridged slides.** Bright-field images showing deyolked embryonic tissue mounted on a bridged coverslip. (A) A deyolked embryo. (B) An embryo was trimmed to obtain the desired tissue (magenta asterisk, used for imaging the gut endoderm). (C) The desired tissue was transferred in PBST to a 24×60 mm bridged coverslip. (D) PBST was removed by a glass pipette (cyan arrowhead), while the tissue was stabilized with a metal probe (magenta arrowhead). (E) The tissue after removal of PBST. (F) The tissue was mounted between the bridged coverslip and a 24×24 mm coverslip, and mounting medium was added, submerging the sample from the bottom left to the top right.

**Fig. 5.**
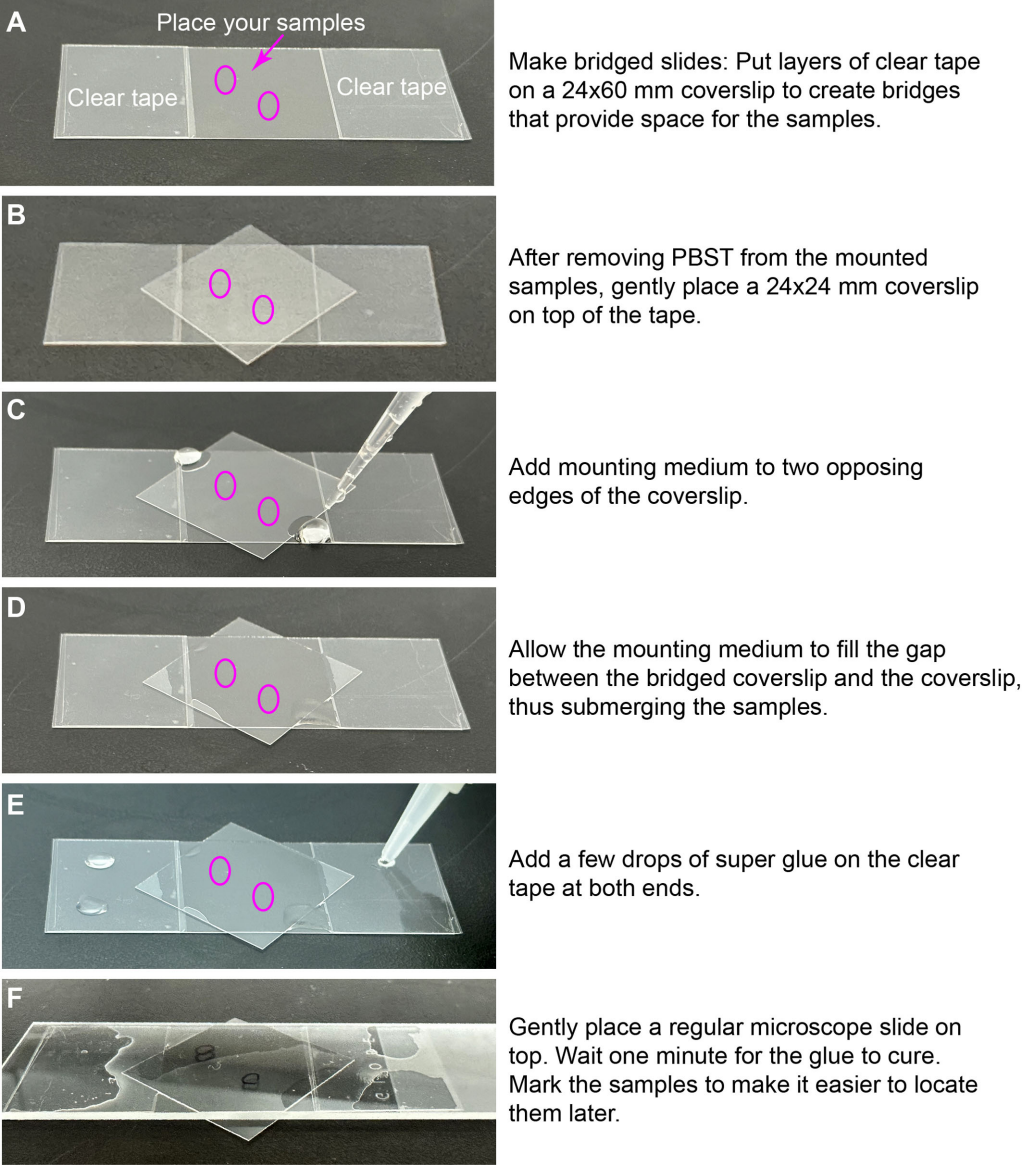
**Deyolked embryonic tissue mounted on bridged slides.** Images showing the series of steps for embryo mounting. Descriptions of each step are provided in the right panels.

Deyolked embryos, with or without immunostaining, can now be prepared for mounting on slides for confocal imaging. However, due to the curvature of the embryo (Fig. 4A), it is not possible to mount the entire embryo flat. Therefore, before mounting, it is necessary to carefully cut the embryonic tissue using forceps to obtain the desired region of interest (Fig. 4B). In this study, the focus is on the gut endoderm located in the posterior region of the embryos (Fig. 4B). Once the desired section is obtained, transfer it along with some PBST onto a specially prepared bridged slide using a glass transfer pipette (Fig. 4C). The bridged slides are created by placing layers of clear tape on both ends of a coverslip (24×60 mm), which creates space for the embryo samples (Fig. 5A). This design prevents the embryonic tissues from being squashed during the mounting process. The number of clear tape layers required depends on the developmental stage of the embryos. Typically, three layers of tape are sufficient for somite-stage embryos, while four layers are recommended for embryos older than 24 hpf.

Once the samples are placed on the slides, it is important to orient them in a way that allows the tissues of interest to be within the working distance of the objective lenses. Since the endoderm tissue is situated on the ventral side of the embryos, which becomes exposed after removing the yolk, we position the ventral side of the tissue facing downwards (while the dorsal side faces upwards) on the bridged coverslip, which interfaces with the microscope objective lens. To achieve the desired orientation of the embryonic tissue, gently manipulate the tissue using a fine metal probe. Carefully move the tissue into the desired position while ensuring that the embryo's orientation is maintained by holding the probe in one hand. And then, using a glass transfer pipette in the other hand, remove as much PBST as possible from the slide (Fig. 4D). It's important to note that when touching the pipette to the PBST, there is a risk of altering the embryo's orientation due to surface tension. In such cases, the embryo may need to be reoriented using the metal probe while the pipette tip is in contact with the PBST. Throughout the process of PBST removal, it may be necessary to readjust the embryonic tissue continuously, which might require adding PBST back onto the slide to facilitate reorientation. Additionally, filter paper strips can be used to remove PBST while using the metal probe to orient the tissue.

After removing the PBST (Fig. 4E), position a 24×24 mm coverslip at a 45-degree angle relative to the sides of the 24×60 mm coverslip, creating spaces in the four corners for the mounting medium (Figs 4F, 5B). Add drops of the mounting medium into two opposing triangular spaces between the 24×24 mm coverslip and the tape (Fig. 5C). The medium will fill the space between the 24×60 mm and 24×24 mm coverslips, ensuring the samples are immersed (Figs 4F, 5D). Once the space between the coverslips is filled with the mounting medium, apply two drops of super glue on each set of tapes (Fig. 5E) and carefully place a regular slide on top (Fig. 5F). Allow the glue to cure for 1 min, then label the samples with a marker to facilitate locating them during imaging (Fig. 5F). The regular slide provides weight and stability to the samples. It is crucial to have this support as any slight disturbance to the square coverslip can damage the sample, and the tension from the oil used in confocal imaging can slightly bend the thin bridged coverslips.

**Fig. 6.**
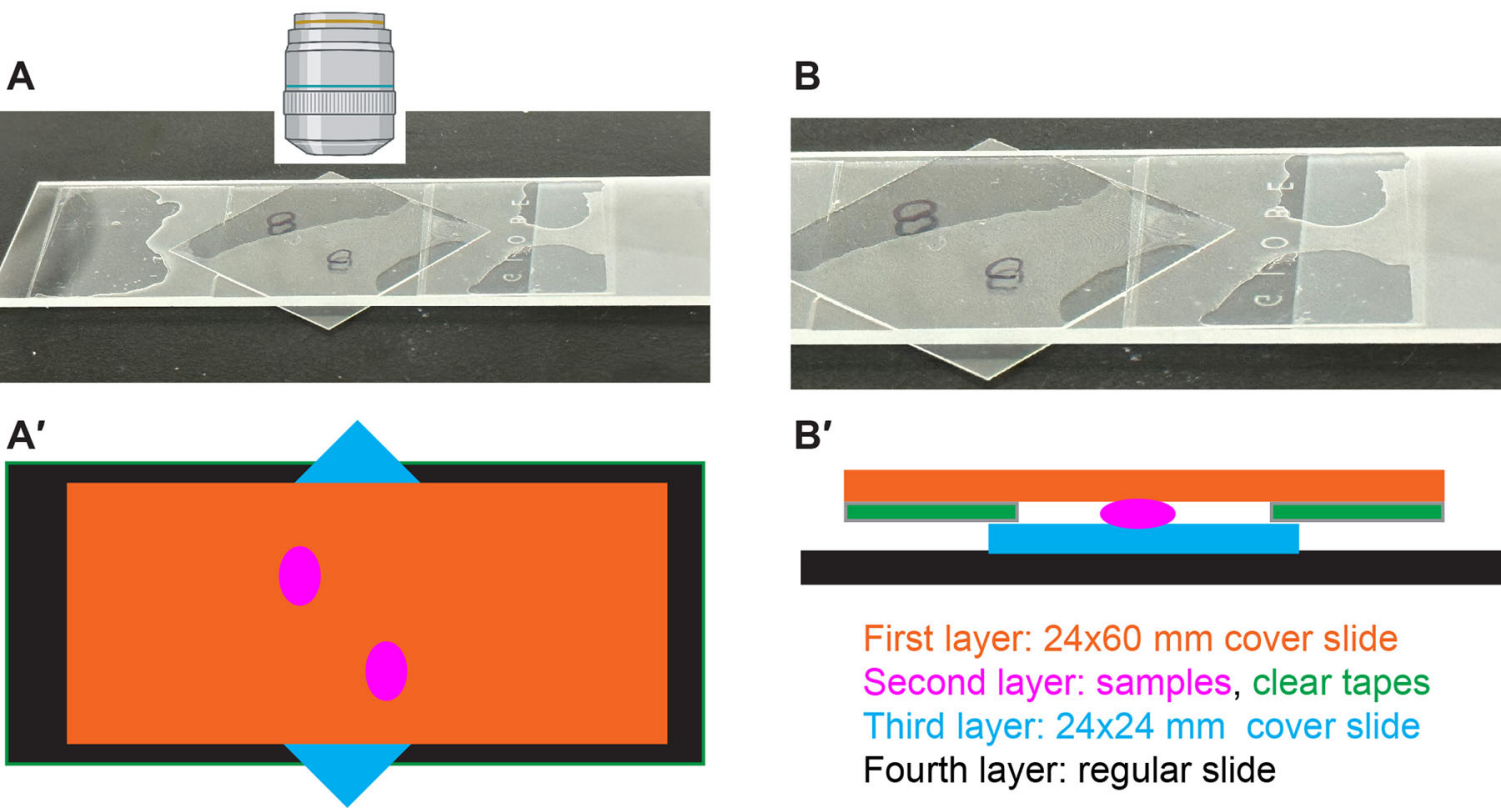
**Samples are placed on a bridged slide for imaging.** (A) Image showing the final slide with samples mounted between the 24×60 mm and 24×24 mm coverslips, supported by a regular slide. (B) Zoomed-in image of A. (A′-B′) Schematic showing the construction of a bridged slide (A′, top view; B′, side view), which consists of multiple layers: the first layer (orange) is a 24×60 mm coverslip, the second layer is made of clear tape (green) and samples (magenta); the third layer is a 24×24 mm coverslip (blue); and the fourth layer is a regular slide (black).

The samples are now securely mounted on the bridged slides, composed of multiple layers: the first layer is the 24×60 mm coverslip, followed by the clear tape with embedded samples, then the 24×24 mm coverslip, and finally the regular slide (Fig. 6). To perform imaging, position the 24×60 mm coverslip facing the microscope objective. It is recommended to image the mounted samples as soon as possible. However, if immediate imaging is not possible, the slides can be stored in a humidified container at 4°C for a maximum of 3 days.

### Imaging conditions

In our studies, we frequently used the following imaging conditions. Initially, we employed a 20× objective lens to capture Z-projection images of a large tissue area, which aided in locating the regions of interest. To minimize photobleaching, we recommend using a medium speed setting (e.g. 8) and larger Z-intervals instead of the optimal settings. For high-resolution imaging, we used an EC Plan-Neo 40×/NA 1.3 oil objective and acquired Z-stacks at optimal intervals using the following settings: 1,024×1,024 pixels, 1.5–2 zoom, 7 speed, and 2 averaging. In the case of thick tissues, we utilized an LD C-Apo 40×/NA 1.1 water objective (Hu et al., 2021).

### Representative results

**Fig. 7.**
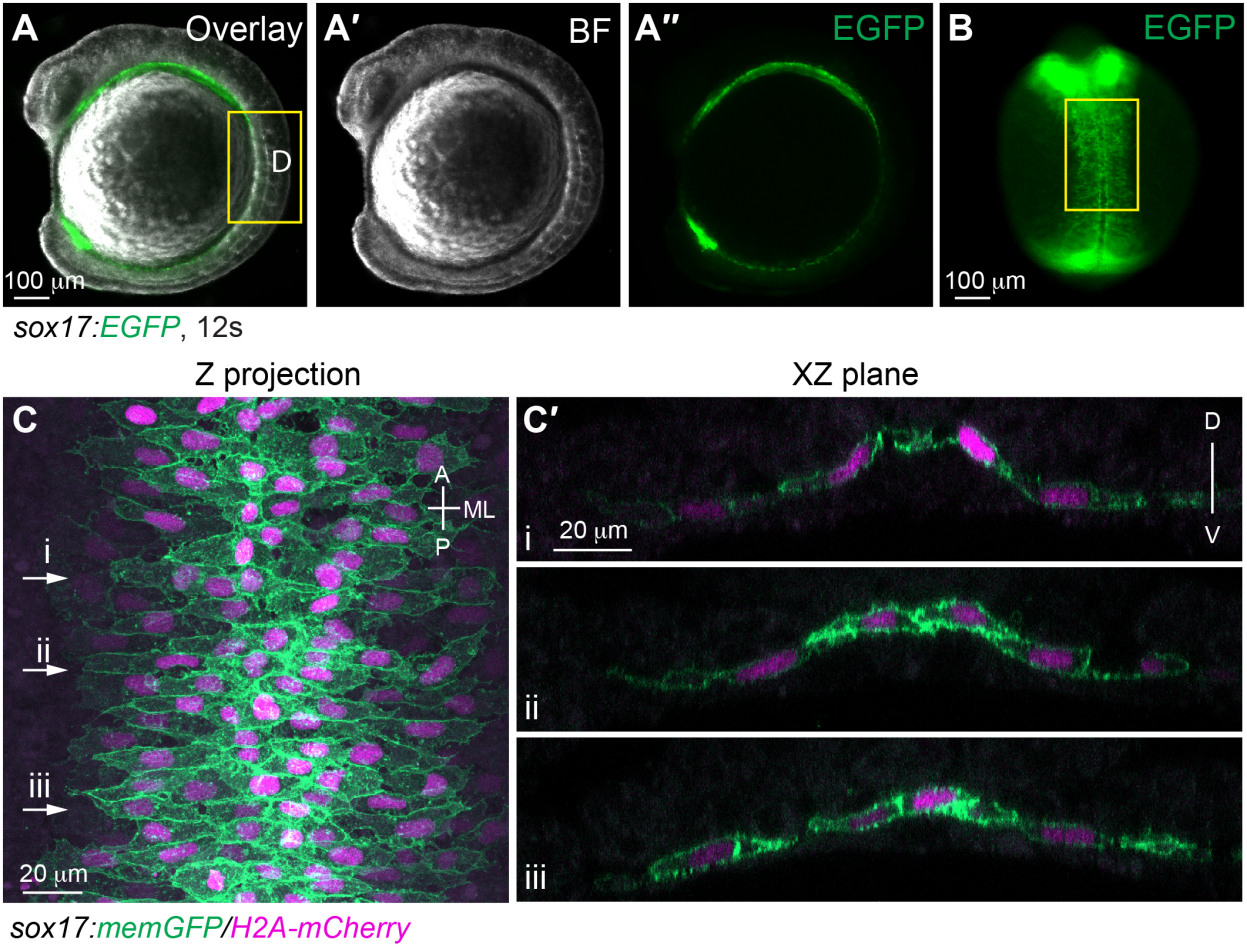
**The morphology of gut endoderm at 12 s.** (A-B) Images of a live embryo in which the endoderm is labeled with EGFP. (A-A″) Lateral view. (A) Overlay image of brightfield (A′) and epifluorescence image of EGFP (A″). (B) Dorsal view. Epifluorescence image of EGFP. (C-C′) Confocal images taken from a flat-mounted sample at a similar region outline in B (highlighted by yellow rectangles), showing endodermal cells labelled with the plasma membrane (GFP) and nuclei (pseudo-colored magenta). (C) Z projection of XY view. (C′) Images of XZ planes taken at the positions marked by i-iii in C. A, anterior; P, posterior; ML, mediolateral; D, dorsal; V, ventral.

**Fig. 8.**
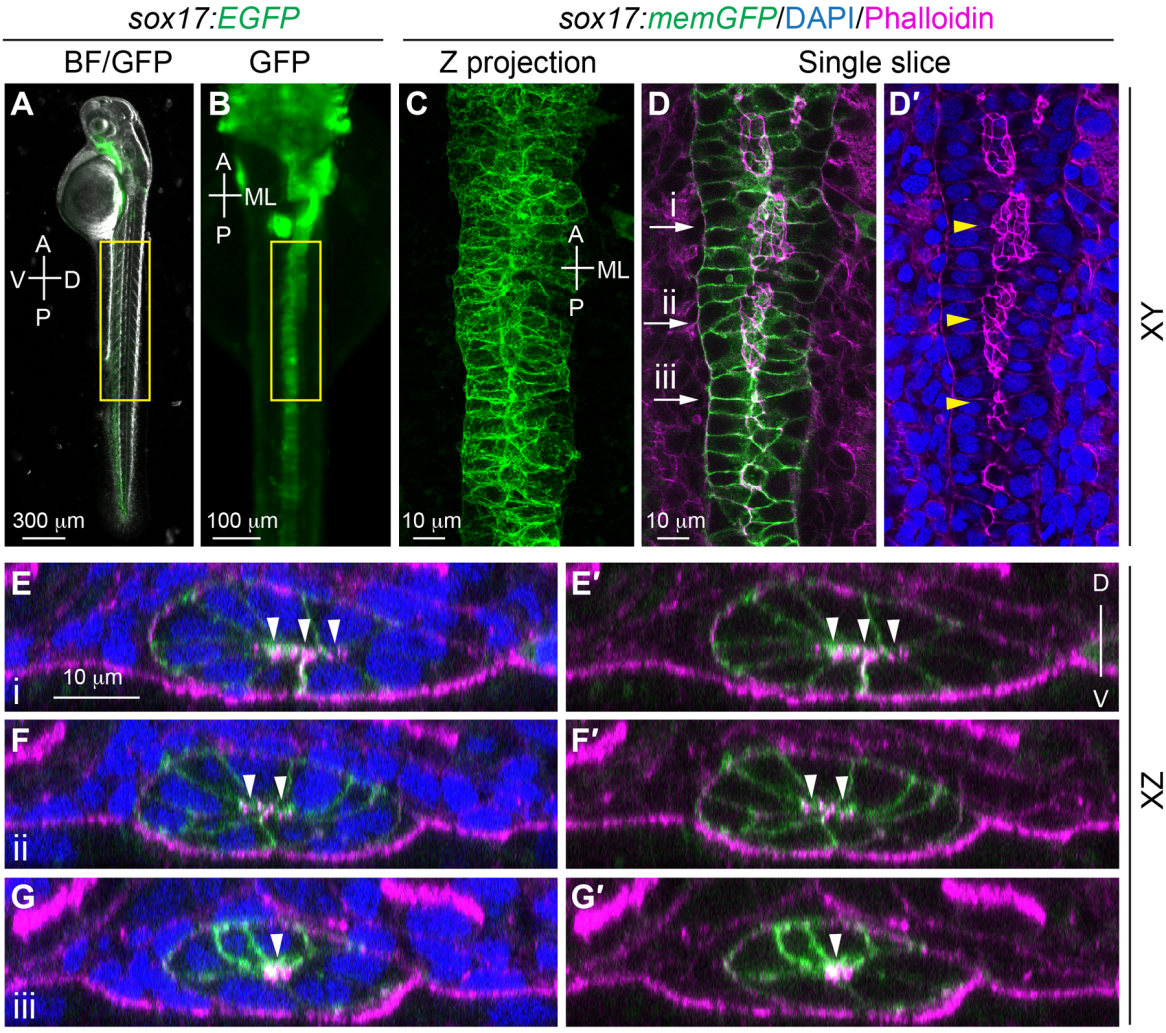
**The morphology of gut endoderm in 48 hpf embryos.** (A-B) Images of a live embryo in which the endoderm is labeled with EGFP. (A) Overlay image of brightfield and epifluorescence of EGFP, Lateral view. (B) Epifluorescence EGFP image. Dorsal view. (C-G′) Confocal images taken from a flat-mounted sample at a similar region outline in B (highlighted by yellow rectangle), showing the expression of actin (detected by Phalloidin staining) in memGFP-labelled gut endoderm. Yellow arrowheads, actin-enriched gut lumen. (C-D′) Images of XY view. (C) Z projection image. (D-D′) Single plane images. (E-G′) Images of XZ planes taken at the positions marked by i-iii in D. White arrowheads, actin enriched sites. A, anterior; P, posterior; V, ventral; D, dorsal; ML; mediolateral.

In zebrafish embryos, the endoderm is situated deep within the embryonic tissues and is in close proximity to the yolk (Figs 7A, 8A). Using the deyolking and mounting techniques outlined earlier, it becomes possible to bring the endoderm closer to the surface and expose it to the objective lens. This facilitates the acquisition of high-quality images, enabling the study of morphogenetic changes occurring during endoderm development.

The anterior part of the endoderm gives rise to endodermal pouches, which are crucial for craniofacial development (Choe and Crump, 2015; Piotrowski and Nusslein-Volhard, 2000). The posterior part of the endoderm, also known as the gut endoderm (Fig. 7A,B, indicated by yellow rectangles), gives rise to the narrow gut tube and associated organs such as the liver and pancreas (Balaraju et al., 2021; Ober et al., 2003; Zorn and Wells, 2009). During gastrulation, endodermal cells exhibit behaviors similar to mesodermal cells, migrating individually and undergoing convergence and extension (C&E) (Mizoguchi et al., 2008; Nair and Schilling, 2008). As segmentation progresses, endodermal cells gradually form cell–cell contacts and organize into a sheet-like structure that continues C&E, narrowing and extending the body axis (Ober et al., 2003; Ye and Lin, 2013). By the stage of 14 somites (14 s), the anterior endoderm sheet narrows to a certain extent, and then undergoes a slight expansion (Ye and Lin, 2013; Ye et al., 2015). The posterior endoderm continues C&E until a narrow rod structure is formed by 18 s (Balaraju et al., 2021; Miles et al., 2017).

Despite the similarities in C&E behavior between endoderm and mesoderm cells in establishing the body plan, the underlying mechanisms are distinct. The Wnt/Planar Cell Polarity (Wnt/PCP) signaling pathway, which is critical for mesodermal and ectodermal cell polarity and migration (Jessen et al., 2002; Topczewski et al., 2001), is not required for the migration of endodermal cells during gastrulation (Mizoguchi et al., 2008; Nair and Schilling, 2008). However, zebrafish embryos carrying mutations in genes involved in the Wnt/PCP pathway exhibit a significantly wider endoderm sheet compared to control embryos, suggesting that Wnt/PCP signaling is involved in C&E of the gut endoderm during segmentation (Balaraju et al., 2021; Miles et al., 2017). During gastrulation, the polarization of mesodermal cells is regulated by Wnt/PCP signaling (Jessen et al., 2002; Topczewski et al., 2001). However, the polarization of endodermal cells during segmentation was not detected in a study that used a transgenic *Tg(sox17:EGFP)* line, in which the endodermal cells are labeled with EGFP (Miles et al., 2017). In this line, GFP is expressed throughout the cytosol, but it does not specifically label the boundaries of the endodermal cells, posing challenges in assessing cell polarity (Miles et al., 2017).

To investigate the cellular morphology of endodermal cells during segmentation, we created a transgenic zebrafish line called *Tg(sox17:memGFP/H2A-mCherry)*, which specifically marks the plasma membrane and nuclei of endodermal cells with GFP and mCherry, respectively (Hu et al., 2021). The endoderm is positioned deep within the embryo, sandwiched between layers of mesodermal and ectodermal tissues as well as the yolk, rendering it visible primarily under low magnification objectives (Fig. 7B). To overcome this limitation, we employed the deyolking method described above, and the endoderm was imaged from the ventral side, enabling high-resolution imaging. Using this technique, we made the novel observation that during early- and mid-segmentation (between the tailbud and 12 s stages), endodermal cells undergo progressive elongation and polarization along the mediolateral axis, spanning the entire endodermal sheet (Fig. 7C) (Balaraju et al., 2021), which is contrary to the results obtained from the *Tg(sox17:EGFP)* line (Miles et al., 2017). During this stage, the endoderm is a monolayer tissue, as demonstrated by the XZ view of images at multiple regions (Fig. 7C′).

Following the 12 s stage, endodermal cells undergo apical constriction and adopt a rod-like structure, which eventually transforms into the gut tube by 48 hpf (Fig. 8A,B) (Balaraju et al., 2021). To examine the organization of the gut tube, we employed the aforementioned yolk removal method and performed staining of the embryos using phalloidin and DAPI. We then mounted the posterior portion of the embryos, which contains the gut tube and associated organs, on bridged slides as described above and performed confocal imaging. We could assess the gut tube from different angles by analyzing the Z-stack images. Single-plane images captured at the central region of the gut tube unveiled the enrichment of actin within the morphology of the central lumen (Fig. 8D,D′). Notably, the gut lumen appeared to narrow gradually from the anterior to the posterior (Fig. 8D,D′), suggesting that the lumen developed first in the anterior region. Corroborating these findings, XZ-view images showed that in the gut tube, cells are arranged as rosettes with actin-enriched central points (Fig. 8E,G′). Using this deyolking method to obtain images of mutants affecting Wnt/PCP signaling, we were able to demonstrate that this signaling is required for the polarity of endodermal cells during early- and mid-segmentation, but not apical constriction, for gut formation (Balaraju et al., 2021).

## DISCUSSION

### Advantages of the deyolking method

The zebrafish is highly transparent, making it amenable to imaging. Modern genetic tools enable the generation of transgenic and knock-in zebrafish lines, which can be used to visualize specific sensors, molecules, cells, and tissues in embryos during development. This allows for the study of cellular and molecular mechanisms at unprecedented levels. However, imaging deep tissues, such as the endoderm, is challenging or even impossible, as most imaging techniques only allow for the visualization of superficial tissues, such as the posterior lateral line primordium (PLLP) (Xu et al., 2012).

To overcome this limitation, we developed the deyolking method, which involves removing the yolk to enable the imaging of tissues from the ventral side, unobstructed by the yolk. The use of bridged slides with adequate space to accommodate tissues prevents squeezing and misshaping, thereby maintaining tissue integrity. This sample preparation method allows for high-resolution imaging, which improves the accuracy and quality of data compared to data acquired at low magnification. For example, using this method, we discovered striking morphogenetic changes in endodermal tissue during development. Imaging embryos at different stages revealed that endodermal cells gradually elongate and polarize mediolaterally during a specific developmental period (Fig. 7). They then undergo apical constriction to transform the monolayer tissue into a gut tube with a rod structure (Fig. 8). Additionally, imaging large tissue areas enabled us to identify differences in tissue organization at different locations. For example, we observed that the lumen of the gut tube gradually increases in size in the anterior regions of this structure, providing an indication of how it is formed (Fig. 8).

However, this method has some limitations. For example, it is not suitable for obtaining dynamic changes *in vivo*, which require time-lapse experiments in live embryos. Additionally, imaging thick and large-scale structures with high resolution using this method can be challenging due to the limitations of objective lenses. To overcome these limitations, other techniques such as selective plane illumination microscopy and light-sheet microscopy may be more suitable (Abu-Siniyeh and Al-Zyoud, 2020; Renninger and Orger, 2013).

### Comparison with other methods

It is common practice to remove the yolk to observe tissue markers’ expression in both the anterior and posterior regions of embryos simultaneously in *in situ* hybridization (ISH) experiments (Cheng et al., 2014). However, the standard ISH method involves fixing embryos in 4% PFA, removing the yolk by hand tools, which is laborious and time-consuming, and residual yolk cells can interfere with obtaining confocal images. Alternatively, the yolk can be removed in live embryos by injecting MP-PNP, a membrane-impermeant non-hydrolyzable analog of ATP, and manually removing the yolk with tungsten microneedles (Langenberg et al., 2003). This method is suitable for dissecting embryo tissues at later stages, and residual yolk cells do not affect imaging certain tissues, such as the brain.

Other methods for imaging deep tissues include cryosectioning and vibratome sectioning. Cryosectioning involves fixing embryos, embedding them in agarose, and freezing them in liquid nitrogen. The frozen blocks are then cut into 10-50 µm thin sections using a cryostat machine and placed on microscope slides for immunostaining or direct imaging (Hu et al., 2018; Ye et al., 2015). Vibratome sectioning involves embedding fixed embryos in agarose and cutting them into 100-200 mm thick sections (Trinh and Stainier, 2004; Ye et al., 2015; Yin et al., 2010). These thick agarose blocks can be placed in a 24-well dish for immunostaining. The angle and the location of cutting are both critical to interpreting the data, and landmarks are normally needed to identify the locations of the sections in the native embryo. Although these methods can provide high-resolution images, they require exceptional skill to obtain sections that are adequate for data analysis. While images of sections can obtain high-quality XZ views, it could be highly useful to combine data from both sectioning and whole-mount samples.

### Optimizing the deyolking method

The procedures described here may need to be optimized when imaging different tissues in zebrafish at different developmental stages. The duration of 1% PFA fixation can be tested to determine the optimal time for the specific tissues being imaged. If the tissues are not easily damaged, more aggressive pipetting can be used to remove the yolk cells more quickly. For embryos older than day 1, the yolk extension has developed, and it needs to be manually removed with sharp forceps. The layers of clear tape used to create the bridged slides can be optimized for different tissues to prevent sample squeezing and damage.

In addition, the deyolking method can be used to image a variety of tissues or organs beyond the endoderm. Because the embryo is flatter on the ventral side facing the yolk, as opposed to the round dorsal side, mounting the deyolked embryo with the ventral side down makes the specimen more stable, allowing for better imaging. Thus, the deyolking method can be used for a wide range of imaging applications and can be further optimized for specific tissues or organs. Additionally, the bridged slides can be used to mount samples from other animal species, such as frog embryos.

## MATERIALS AND METHODS

### Zebrafish strains and maintenance

Zebrafish were maintained using animal protocols that were approved by the University of Iowa Animal Care and Use Committee. Embryos were obtained by natural spawning and staged according to morphological criteria or hours post fertilization (hpf) at 28.5°C or specified temperatures, as described previously (Kimmel et al., 1995). The following zebrafish lines were used: AB*/Tuebingen, *Tg(sox17:EGFP)* (Mizoguchi et al., 2008), *Tg(sox17:memCherry)* (Ye et al., 2015), *Tg(sox17:memGFP/H2A-mCherry)* (Hu et al., 2021).

### Equipment

The following equipment is used: Leica DMI 6000 microscope with a 5×/NA 0.15 objective (Leica microsystem, Wetzlar, Germany); Leica M165FC stereomicroscope with a Leica DFC290 Color Digital Camera; Zeiss inverted LSM880 or LSM980 laser microscope (Carl Zeiss, Hebron, KY, USA); HIC Kitchen Double-Ear Fine Mesh Strainer, White, 3-Inch (through Amazon); Pipette pump (SP Bel-Art; F37898-0000; Wayne, NJ, USA); Pasteur pipette (Avantor through VWR International; 53283-916; Radnor, PA, USA); Disposable transfer pipette (Dot Scientific; P4122-01; Burton, MI, USA); Dumont #5 forceps (Fine Science Tools; 11252-20; Foster City, CA, USA); 9-well depression plate (PYREX, Fisher Scientific; 722085 or 13-748B; Corning, NY, USA); Glass dish (Thermo Fisher Scientific, 08-747A; Waltham, Massachusetts, 02451, USA); 24-well plate (USA Scientific; CC7682-7524; Ocala, FL, USA); 24×60 mm cove slip (Leica Biosystems; 3800160; Nussloch, 69226, Germany); Scotch clear tape; 24×24 mm coverslip (Synthware Glass, Kemtech America; 0340-1650; Easton, PA, USA); Microscope slide (Globe Scientific; 1380-20; Paramus, NJ, USA).

### Reagents

The following reagents are used: Instant Ocean sea salt (through Amazon); Paraformaldehyde (Thermo Fisher Scientific; AC416780250; Waltham, MA, USA); Triton X-100 (Thermo Fisher Scientific); Acetone (Thermo Fisher Scientific); Dimethyl Sulfoxide (DMSO) (Thermo Fisher); DAPI (0.2 mg/ml, D1306, Invitrogen through Fisher Scientific); Phalloidin (1:100, A12380, Invitrogen through Fisher Scientific); Glycerol (ACS grade 99-100% purity).

### Recipes

The following are recipes for solutions and buffers used: egg water: Instant Ocean sea salt in ddH2O (60 µg/mL); Phosphate-buffered saline (PBS): 137 mM NaCl, 2.7 mM KCl, 10 mM Na_2_HPO_4_, 1.8 mM KH_2_PO_4_. Adjust to pH 7.4; 4% paraformaldehyde in PBS; 10% Triton X-100: Pour 100% Triton-100 slowly into a 50 mL tube to reach to reach the 5 mL mark, add 45 mL H2O. Mix well on a rotator; AB blocking buffer: 5% serum (goat or sheep) and 5 mg/ml BSA (from 100 mg/ml) in PBST-DMSO; PBST-DMSO: PBST with 2% DMSO (freshly prepared, lasts 1 week); 20% n-propyl gallate (weight/volume) (0.2 g n-propyl gallate in 1 ml of 100% DMSO); Mounting medium (90% Glycerol, 0.2% n-propyl gallate in PBS).

## Supplementary Material

10.1242/biolopen.060048_sup1 Supplementary information
