# Supplementary material for: Flat mount preparation for whole-mount fluorescent imaging of zebrafish embryos
Source: Biol Open. 2023 Jul 20;12(7):bio060048. doi: 10.1242/bio.060048 (PMC10373579; doi:10.1242/bio.060048)
Supplement: Supplementary information [file biolopen-12-060048-s1.pdf]

Table S1. Antibodies that worked in zebrafish embryos using the described fixative method.

Antibody	Target	Company	Catalog #	Dilution	Reference
anti-GFP	GFP	Santa Cruz	sc-8334	1:200	[1, 2]
anti-ZsYellow	ZsYellow	Origene	A180004	1:200	[1]
anti-Kaede	Kaede	MBLI	2F4	1:200	[1]
anti-ZO1	ZO1	Invitrogen	33-9100	1:200	[1, 3]
anti-Fibronectin	Fibronectin	Sigma	F3648	1:300	[1, 3-5]
anti-PKCζ	PKCζ	Santa Cruz	C-20	1:200	[1]
anti-β-catenin	β-catenin	Sigma	C7207	1:200	[4]
anti-MF20	Myosin heavy chain	DSHB		1:200	[3]
anti-S46	atrium-specific Myosin heavy chain 6	DSHB		1:200	[3]
anti-Gβ	G protein b subunit	Santa Cruz	Sc-166249	1:200	[6]
anti-GNB1	G protein b subunit 1	GeneTex	GTX114442	1:200	[6]
anti-laminin	laminin	ThermoFisher Scientific	RB-082-A	1:300	[4]
anti-N-cadherin	N-cadherin	Abcam	ab211126	1:200	[7]
anti-Eea-1	Eea-1	Abcam	ab2900	1:300	[7]
anti–C-MYC	C-MYC	DSHB	9E10	1:200	[2]

Reference:

1. Ye, D., et al., *Endoderm convergence controls subduction of the myocardial precursors during heart-tube formation.* Development, 2015. **142**(17): p. 2928-40.
2. Hu, B., et al., *Glypican 4 mediates Wnt transport between germ layers via signaling filopodia.* J Cell Biol, 2021. **220**(12).
3. Xie, H., et al., *S1pr2/Galpha13 signaling regulates the migration of endocardial precursors by controlling endoderm convergence.* Dev Biol, 2016. **414**(2): p. 228-43.
4. Hu, B., et al., *Glypican 4 and Mmp14 interact in regulating the migration of anterior endodermal cells by limiting extracellular matrix deposition.* Development, 2018. **145**(17).
5. Gao, Y., et al., *Fibronectin and Integrin alpha5 play overlapping and independent roles in regulating the development of pharyngeal endoderm and cartilage.* Dev Biol, 2022. **489**: p. 122-133.

Biology Open • Supplementary information

6. Ke, W., et al., *Gbeta1 is required for neutrophil migration in zebrafish.* Dev Biol, 2017. **428**(1): p. 135-147.

7. Balaraju, A.K., et al., *Glypican 4 regulates planar cell polarity of endoderm cells by controlling the localization of Cadherin 2.* Development, 2021. **148**(14).

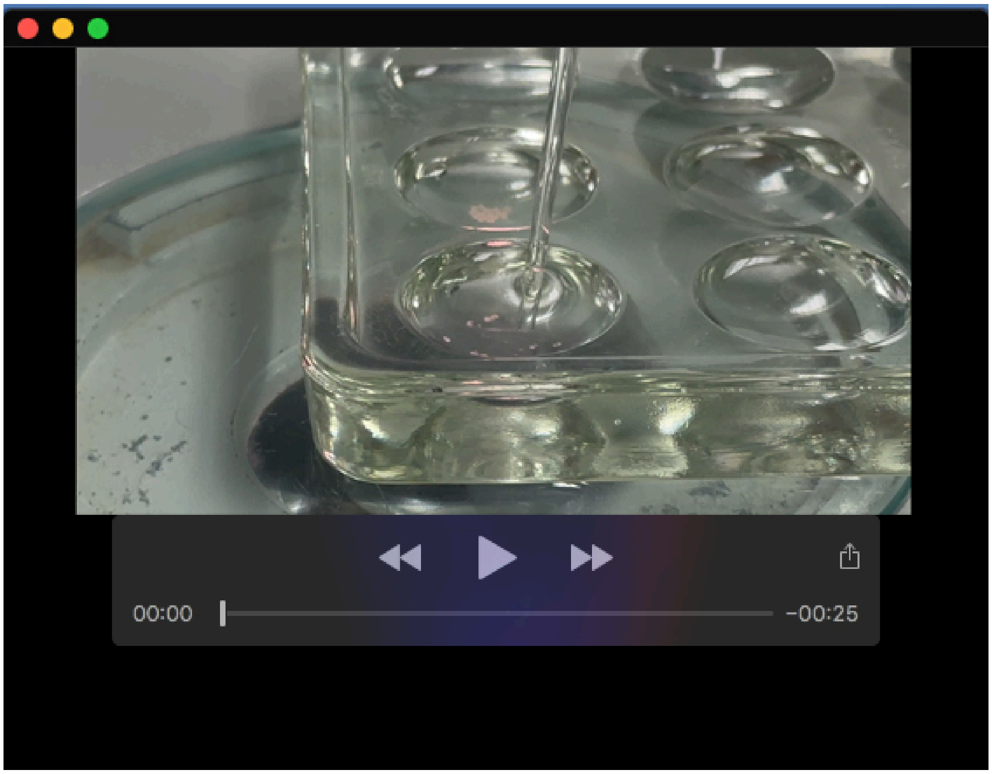

Movie 1. Embryonic yolks are manually removed by pipetting. Fixed embryos are placed in a 9-well depression dish containing ice-cold PBST. After the yolk sac is pierced, embryos are pipetted up and down by a glass transfer pipette to create forces to remove the yolk.
